# Bone Marrow Mesenchymal Cells Improve Muscle Function in a Skeletal Muscle Re-Injury Model

**DOI:** 10.1371/journal.pone.0127561

**Published:** 2015-06-03

**Authors:** Bruno M. Andrade, Marcelo R. Baldanza, Karla C. Ribeiro, Anderson Porto, Ramon Peçanha, Fabio S. A. Fortes, Gisele Zapata-Sudo, Antonio C. Campos-de-Carvalho, Regina C. S. Goldenberg, João Pedro Werneck-de-Castro

**Affiliations:** 1 Laboratório de Biologia do Exercício, Instituto de Biofísica Carlos Chagas Filho e Escola de Educação Física e Desportos, Universidade Federal do Rio de Janeiro, Rio de Janeiro, RJ, Brasil; 2 Instituto de Biofísica Carlos Chagas Filho, Centro de Ciências e Saúde, Universidade Federal do Rio de Janeiro, Bloco G, Ilha do Fundão, Rio de Janeiro, RJ, Brasil; 3 Departamento de Farmacologia Básica e Clínica, Universidade Federal do Rio de Janeiro, Centro de Ciências e Saúde, Bloco J, Ilha do Fundão, Rio de Janeiro, Brasil; National Institutes of Health, UNITED STATES

## Abstract

Skeletal muscle injury is the most common problem in orthopedic and sports medicine, and severe injury leads to fibrosis and muscle dysfunction. Conventional treatment for successive muscle injury is currently controversial, although new therapies, like cell therapy, seem to be promise. We developed a model of successive injuries in rat to evaluate the therapeutic potential of bone marrow mesenchymal cells (BMMC) injected directly into the injured muscle. Functional and histological assays were performed 14 and 28 days after the injury protocol by isometric tension recording and picrosirius/Hematoxilin & Eosin staining, respectively. We also evaluated the presence and the fate of BMMC on treated muscles; and muscle fiber regeneration. BMMC treatment increased maximal skeletal muscle contraction 14 and 28 days after muscle injury compared to non-treated group (4.5 ± 1.7 vs 2.5 ± 0.98 N/cm^2^, p<0.05 and 8.4 ± 2.3 vs. 5.7 ± 1.3 N/cm^2^, p<0.05 respectively). Furthermore, BMMC treatment increased muscle fiber cross-sectional area and the presence of mature muscle fiber 28 days after muscle injury. However, there was no difference in collagen deposition between groups. Immunoassays for cytoskeleton markers of skeletal and smooth muscle cells revealed an apparent integration of the BMMC within the muscle. These data suggest that BMMC transplantation accelerates and improves muscle function recovery in our extensive muscle re-injury model.

## Introduction

Skeletal muscle (SKM) injuries are very commonly diagnosed in sports medicine and, more important, the incidence of re-injury remains very high [1]. The risk of recurrent injury is associated with the extent of residual scar tissue in the muscle [2] induced by a previous damage. The SKM healing process comprises degeneration and inflammation, regeneration, and fibrosis in a complex and well-orchestrated series of events [3,4]. SKM regenerative capacity is due to the presence of a tissue-specific population of myogenic stem cells termed satellite cells, so-called due to its peripheral location on the skeletal muscle myofibre where it lies between the sarcolemma of the myofibre cell and its surrounding basal lamina [5].

However, depending on the severity and frequency of the injury, the regeneration process of skeletal muscle is very slow and incomplete, resulting in strength loss, fibrosis and a high rate of re-injury at the site of the prior injury [3]. Fibrosis is triggered mainly by the expression of transforming growth β (TGF- β) which stimulates fibroblast proliferation and collagen synthesis; and, concomitantly, inhibits both satellite cell proliferation and differentiation into myotubes [6,7]. Therefore, complete recovery of injured skeletal muscle appears to be hindered by fibrosis and usually leads to incomplete muscle healing [3]. The resultant disorganized scar tissue that often replaces damaged myofibers may contribute to muscle injuries recurrence [8].

Therapeutic intervention to treat skeletal muscle injury is controvertial. Common treatments for SKM injuries are RICE (rest, ice, compression, and elevation), nonsteroidal anti-inflammatory drugs, pulsed ultrasound and immobilization. However, none of them treats the main problem, e.g. SKM cell loss and scar tissue formation. Also, routine treatments do not improve satellite cells number, proliferation and differentiation. Therefore, efforts to develop treatments to promote faster and more complete recovery after muscle injury should be focused on the enhancement of muscle regeneration and/or the prevention of fibrosis.

Because fast and complete repair of the injured muscle is the obvious target, especially in athletes, attempts should be made to find clinically feasible modalities to enhance the proliferation phase of muscle regeneration. Cell transplantation has been used to overcome the loss of cells induced by lesions in many organs such as liver [9]^,^, heart [10,11] and skeletal muscle [12,13]. Furthermore, in SKM injuries, a variety of cell types have been tested: myoblasts, which is the most used cell type [14], muscle-derived stem cells [15], hematopoietic stem cells [16,17], bone marrow-derived human mesenchymal stem cells [18], adipose-derived stem cells [13] and BMMC [12,19]. BMMC have been reported to differentiate in vitro into contractile myotubes or convert into myogenic linage in vivo in response to physiological stimuli [17,20–22].

The search for new therapeutic alternatives requires the development of adequate animal models of muscle lesion. In this perspective, the availability of an animal model that mimics the characteristics of human muscle injury is crucial to study the pathophysiological mechanisms of regeneration and possible therapies. Many animal models of muscle injury have been proposed such as injection of cardiotoxin [23,24], mdx transgenic mice, crioinjury, manual clamping, laceration, strain and induced by exercise but none reproduces re-injuries which are the most common type of damage in the orthopedic field.

Thus, the main purpose of this study was to investigate whether BMMC injected directly into damaged muscles could improve muscle function in a rat model with repetitive muscle injuries.

## Methods

### Animals

Male inbred Wistar rats (200–250g) were obtained from the Centro de Pesquisa Gonçalo Muniz (Fiocruz-Bahia/Brasil). Animals were housed at controlled temperature (23°C) with daily exposure to a 12:12-h light-dark cycle and free access to water and standard rat chow. Isogenic rats were used as donors and recipients of bone marrow-derived stromal cells. The Rio de Janeiro Federal University Institutional Committee for Animal Use in Research (CEUA—CCS—EEFD 07) approved this study, which was in accordance with the International Guiding Principles for Biomedical Research Involving Animals (Geneva, Switzerland). The surgery was done under anesthesia with ketamine (50 mg/kg, i. p.) and xylazine (5mg/kg, i. p.) and the animals were killed by cervical dislocation after being anesthetized. All efforts were made to minimize suffering.

### Muscle re-injured model

Animals were anesthetized with ketamine (50 mg/kg) and xylazine (5 mg/kg ip). An incision was made in the skin overlying the lateral portion of the gastrocnemius muscle. The soleus muscle of the right limb was exposed along its entire length and lacerated (crunched 40 times) through 50% of its width and 100% of its thickness with a surgical blade type Basket (0.6mm). Twenty crunches were repeated 7 and 14 days after the first surgical procedure. The contralateral soleus was used as control muscle.

### Cell isolation and culture procedures

The isolation and primary culture of BMMC from femoral and tibial bones of donor rats were performed as described by Fidelis-de-Oliveira et al. [25]. BMMC were cultured in high glucose DMEM supplemented with 10% fetal bovine serum (GIBCO-BRL), 2 mM L-glutamine (Sigma), and antibiotics (100 U/ml penicillin G and 100 μg/ml streptomycin; GIBCO-BRL). BMMC were grown in 75 cm^2^ flasks and maintained at 37°C in a 5% CO_2_ incubator for 1 week during which medium was changed, at least twice, washing away all floating hematopoietic cells. At ~ 80–90% confluence, the cells were detached from the culture flasks with 0.25% trypsin-EDTA (Sigma) and replated. After the third replating, BMMC were resuspended in DMEM without serum, and labeled with 5μg/ml Hoescht 33342 (Sigma- Aldrich) for 20 minutes. The cells were rinsed and centrifuged six times in balanced salt solution (BSS) to remove unbound Hoescht 33342 and kept in warm DMEM for a few minutes before transplantation. This labeling procedure was very efficient, ensuring ~90–95% labeling of cell nuclei. Cell viability was tested after labeling and was never lower than 95%. These cells are capable of differentiation in osteoblasts and adypocites.

### Cell transplantation and experimental groups

Three days after the injury protocol the rats were randomized for treatment with BMMC or vehicle (non-treated group). Under anesthesia, the soleus muscle was exposed and 3 X 10^6^ labeled BMMC were injected with a tuberculin syringe along the border and the center of the scar tissue. To improve retention, cells were suspended in 40 μl of a three-dimensional gel of reconstituted basement membrane (Matrigel Matrix BD Biosciences, USA) and BSS in a proportion of 1:3 [10,13]. Non-treated rats received only Matrigel in BSS (vehicle). After treatment, the leg was stitched closed, and the animals were monitored for 48hs.

### Maximal tension developed in isolated soleus muscle

Fourteen and twenty-eight days after the last injury, BMMC (14d n = 6 / 28d n = 9) and non-treated rats (vehicle- 14d n = 6/ 28d n = 7) rats were anesthetized with isoflurane and soleus muscles were dissected for isometric tension recording. Muscles were mounted in vertical chambers filled with Ringer solution (NaCl 135 mM, KCl 5.0 mM, MgCl_2_ 1.0 mM, Na_2_HPO_4_ 1.0 mM, NaHCO_3_ 25 mM, CaCl_2_ 1.25 mM, glucose 5 mM) and continuously oxygenated with carbogen gas (95% O_2_ /5% CO_2_) at 37°C. One muscle end was attached to a force transducer (Grass, model FT-03) and the other end to a hook fixed at the bottom of the experimental chamber. Soleus muscles were field stimulated (Grass S88) at a rate of 0.2 Hz with pulses of 2 ms duration. The transducer’s electrical signal was amplified (Cyberamp, Axon Instruments), digitized (Digidata 1200, Axon Instruments), displayed and stored on a computer using Axoscope software (Axon Instruments). After stabilization (30 min), maximal isometric tension was obtained by stimulation at 50 Hz. The amplitude of maximal contractile response was normalized to the cross-sectional area (CSA) of the muscle using the equation: Tension (N/cm^2^) = Force/CSA. CSA (cm2) was obtained by the formula: weight (g)/length (cm) * 1.06 g/cm3 (muscle density) (54).

### Tissue architecture and collagen deposition

Soleus muscles were fixed in 10% buffered formalin and embedded in paraffin. Longitudinal and cross sections of 5μm thickness were obtained and mounted on glass slides. Slides were dewaxed and rehydrated with distilled water. Thereafter the samples were stained with heamatoxilin and eosin to provide an overall view of the tissue and with picrosirius stain for collagen deposition evaluation. For the picrosirius staining procedure the slides were incubated in 0.2% Phosphomolybdic acid (Sigma-Aldrich Ltd) for 1 minute following incubation at room temperature for 90 minutes in sirius red solution (0.6g in 600 mL of saturated picric Acid), (Sigma-Aldrich Ltd). The slides were then incubated in 0.01N Hydrochloric Acid (Sigma-Aldrich Ltd) for 2 minutes, dehydrated, cleared in xylene, and mounted with Entellan. Slides were examined in a light Axiovert 100 microscope (Zeiss). For fibrosis quantification (BMMC, 14d n = 3 / 28d n = 4 and vehicle, 14d n = 6 / 28d n = 5), 20 random fields (20X original magnification) were taken. The acquisition was standardized using ImagePro 5.0 and no modifications in the images were done after acquisition. Images were analyzed using ImagePro 5.0 to quantify the red area and the total area. The area of fibrosis was considered the ratio between the red area / total area.

### Centronucleated fibers and fiber cross sectional area quantifications

In order to quantify skeletal muscle hypertrophy, we measured muscle fiber cross-sectional area (BMMC, n = 4 and non-treated, n = 3) 28 days after muscle injury as previously described [26]. Hematoxylin and eosin stained images taken on the microscope were analyzed on Image J program and the area of 500 fibers per soleus was measured. We counted the number of centronucleated regenerating myofibers at 28 days after muscle injury (BMMC, n = 4 and non-treated, n = 3) in 10^3^ fibers as previously described [13]. At least ten fields were analyzed for each muscle, and the average number of regenerating myofibers per 10^3^ total fibers was compared between groups. Nuclei with no discernible surrounding cytoplasm were discarded.

### Immunohistochemistry/Identification of cell transplanted

Soleus muscles were taken from BMMC (n = 5) and non-treated (n = 3) animals 28 after repeated injuries. In order to perform immunohistochemistry, muscles were cryoprotected, cooled in liquid nitrogen, and 10μm consecutive cryostat sections were obtained. Tissue sections were stained with antibodies against smooth muscle myosin heavy chain (MHC (G-4)1:100—Santa Cruz Biotechnology—sc-6956) and troponin I (1:100—clone 5C5, Sigma). Goat anti-mouse secondary antibodies were used as follows: IgG FITC conjugate (1:50—catalog no. F-2012, Sigma) and IgM FITC conjugate (1:50—catalog no. F-9259, Sigma). Thereafter, sections were washed with PBS and mounted with PPD solution (89% glycerol, 10% PBS and 1% p-phenylene diamine—PPD). Grafted BMMC were identified by Hoescht 33342-labeled nuclei excited at 351 nm by UV laser. An Axiovert 135 microscope coupled to a high-resolution Axiocam HR charge-coupled device videocamera (Zeiss) was used to obtain differential interference contrast and fluorescence images from regions of the injured soleus muscle displaying nuclear and cytoskeletal markers. Images were overlaid and processed using Photoshop 7 software (Adobe).

### Statistical analyses

Values are presented as mean ± SD. Differences between the BMMC-treated, vehicle-treated and the contralateral normal muscles in all time points were evaluated by one-way ANOVA followed by Tukey´s post hoc test. Values were considered different when P<0.05.

## Results

### Muscle function

**Fig 1:**
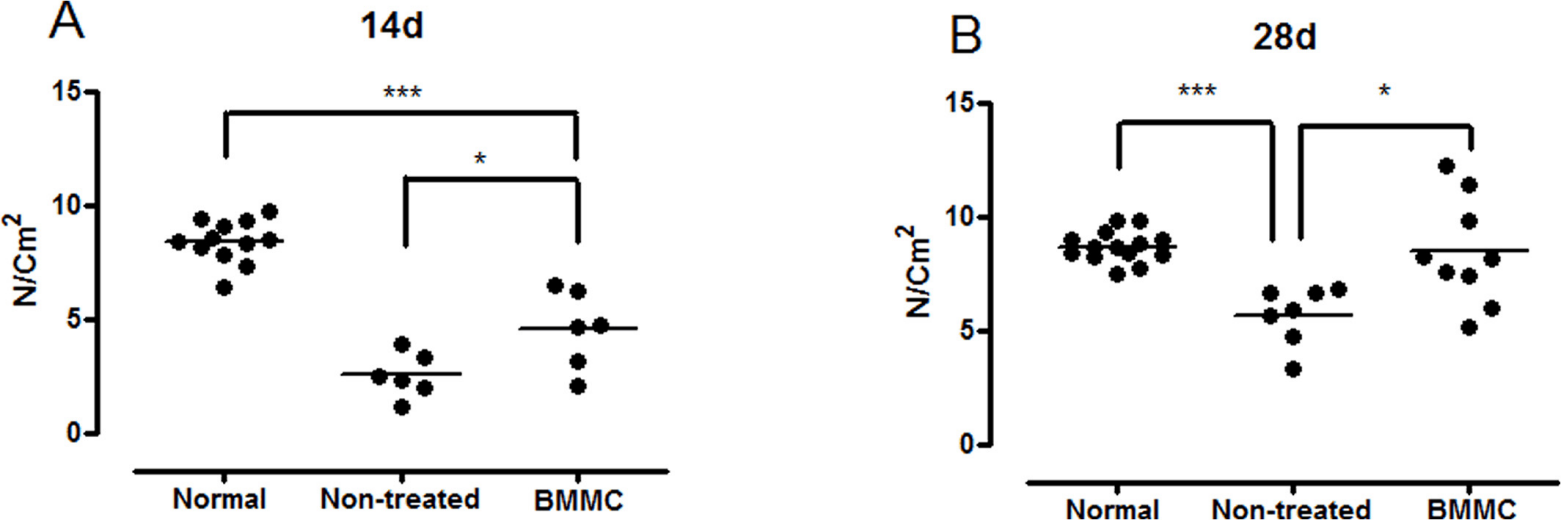
Maximal isometric tension of soleus muscle after BMMC treatment. **(A)** Maximal isometric force in normal (non-injured), BMMC and vehicle-treated groups 14 days after injury. **(B)** Same as in (A) except that animals were analyzed 28 days after injury. Normal group values are from contralateral non-injured muscle. Values are means ± SD. *** p<0.001 * p<0.05. BMMC (14d n = 6 and 28d n = 9) and vehicle-treated rats (14d n = 6 and 28d n = 7)

Our repetitive injury model resulted in great muscle function impairment. Soleus isometric developed force decreased ~70% at day 14 (2.5 ± 0.98 N/cm^2^, p<0.001; Fig 1A) and remained ~33% below non-injured muscles at day 28 (5.7 ± 1.3 N/cm^2^, p<0.001; Fig 1B) days after injury, when compared with the contralateral uninjured muscle (8.4 ± 0.95 N/cm^2^). BMMC treatment increased muscle force at both 14 (4.5 ± 1.7 vs. 2.5 ± 0.98 N/cm^2^, p<0.01) and 28 (8.4 ± 2.3 vs. 5.7 ± 1.3 N/cm^2^, p<0.001) days after injury compared to the vehicle group (non-treated). Moreover, no statistical difference was found between the BMMC-treated and normal muscles at day 28 (8.4 ± 2.3 vs. 8.7 ± 0.68 N/cm^2^, respectively; Fig 1A). These results demonstrate that BMMC can improve muscle function even after severe damage produced by the repetitive injury protocol employed.

### Collagen content, number of centronucleated myofibers and soleos cross-sectional area

**Fig 2:**
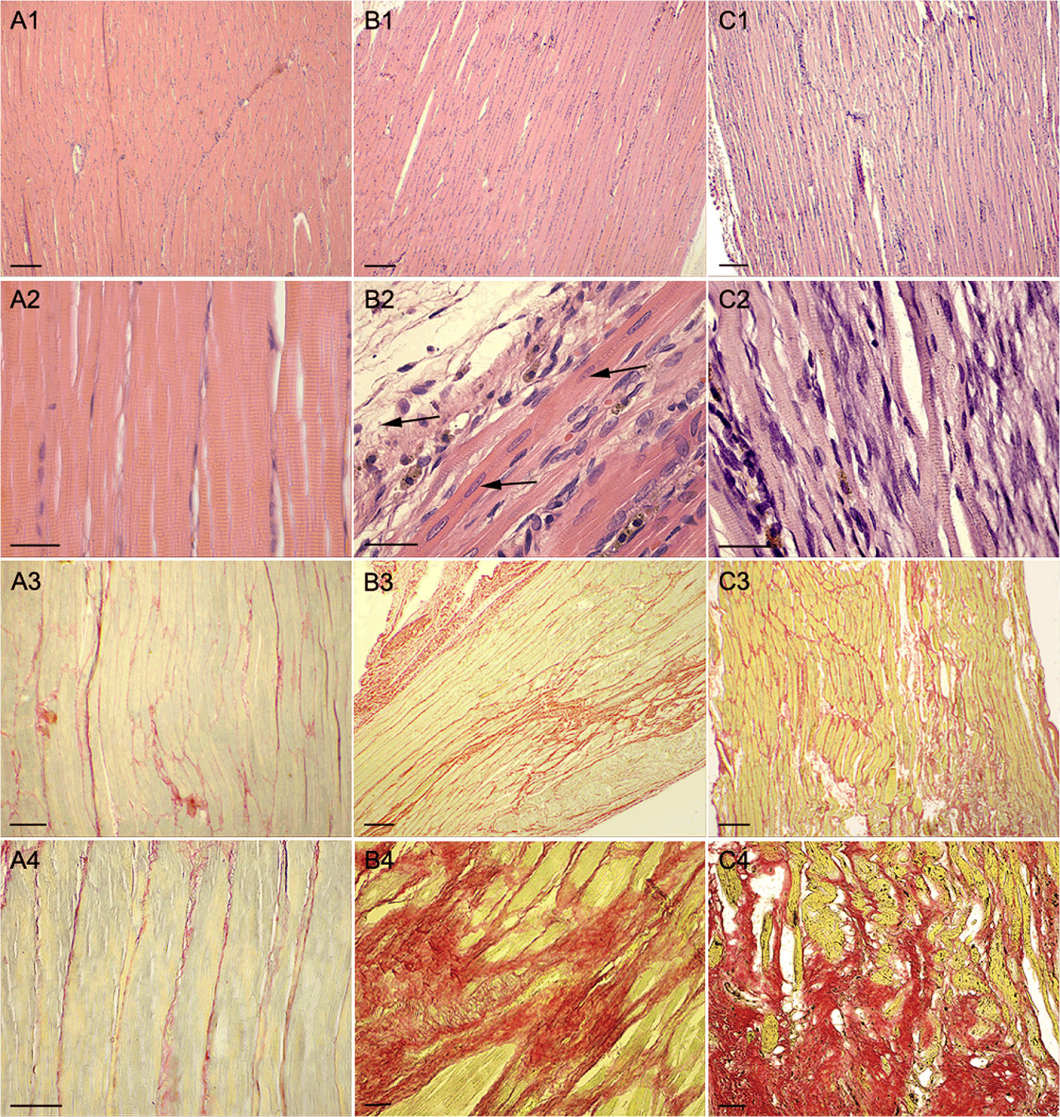
Histological sections stained by heamatoxylin & eosin. (A1) H&E staining image of a rat non-injured soleus muscle 28 days after surgery; (A2) same as in A1 except that vehicle-treated muscle is shown; (A3) same as in A1 but BMMC-treated animal is presented; (A2, A3 and A4) Details of A1, A2 and A3 animals are shown in greater magnification, respectively; (A3) Picrossírius staining image of a rat non-injured soleus muscle 28 days after surgery;; (B3) same as in A3 except that vehicle-treated muscle is shown; (C3) same as in A3 but BMMC-treated animal is presented and (A4, B4 and C4) Details of A3, B3 and C3 animals are shown in greater magnification, respectively; Histological analysis reveals that both groups submitted to muscle injury present wide spread collagen deposition (A3-C4) independent of treatment. Note the central aligned disposition of the muscle fiber nuclei indicating recent muscle fiber regeneration (arrows in B2). Bars correspond to 300 μm in A1-C1 and A3-C3 and correspond to 50μm in A2-C2 and A4-C4.

Soleus muscle collagen content and the presence of transplanted BMMC were carefully examined in postmortem histological evaluation. Normal muscles presented parallel fibers with multiple nuclei located in the periphery and a perfect arrangement of myofibrils giving rise to muscle striations (Fig 2A1 and 2A2). Twenty-eight days after injury, although the vast majority of the muscle presented as normal muscle tissue arrangement, few areas with disarranged tissue organization, sites of inflammation and fibers with centralized nuclei were still present in the vehicle group (Fig 2B1 and 2B2) and BMMC-treated groups (Fig 2C1 and 2C2).

Quantitative analysis of picrosirius staining revealed a significant increase in the amount of fibrosis in both groups at both time points when compared to control muscle (Table 1). This fibrosis was predominantly perivascular and interstitial in most of the tissue of both groups (Fig 2A3–2C4). There was no difference in the amount of collagen deposition after 28 days of treatment in vehicle- and cell-treated groups (Fig 2 and Table 1). We also assessed collagen deposition on soleus cross sections and still found no difference between BMMC and non-treated groups (0.26 ± 0.06 vs 0.31 ± 0.08, respectively).

**Table 1:** Quantification of collagen content.

14 days			28 days		
Normal (n=9)	Non-treated (n=6)	BMMC (n=3)	Normal (n=9)	Non-treated (n=5)	BMMC (n=4)
0.14 ± 0.02	0.34 ± 0.04***	0.29 ± 0.03***	0.14 ± 0.02	0.30 ± 0.04***	0.26 ± 0.04***

Quantification of collagen content in longitudinal sections by picrosirius staining in BMMC-treated and non-treated groups soleus muscle 14 and 28 days after injury. Normal group values are contralateral non-injured muscle. Values are means ± SD of collagen area/total area ratio.

*** p<0,001 compared to normal.

**Fig 3:**
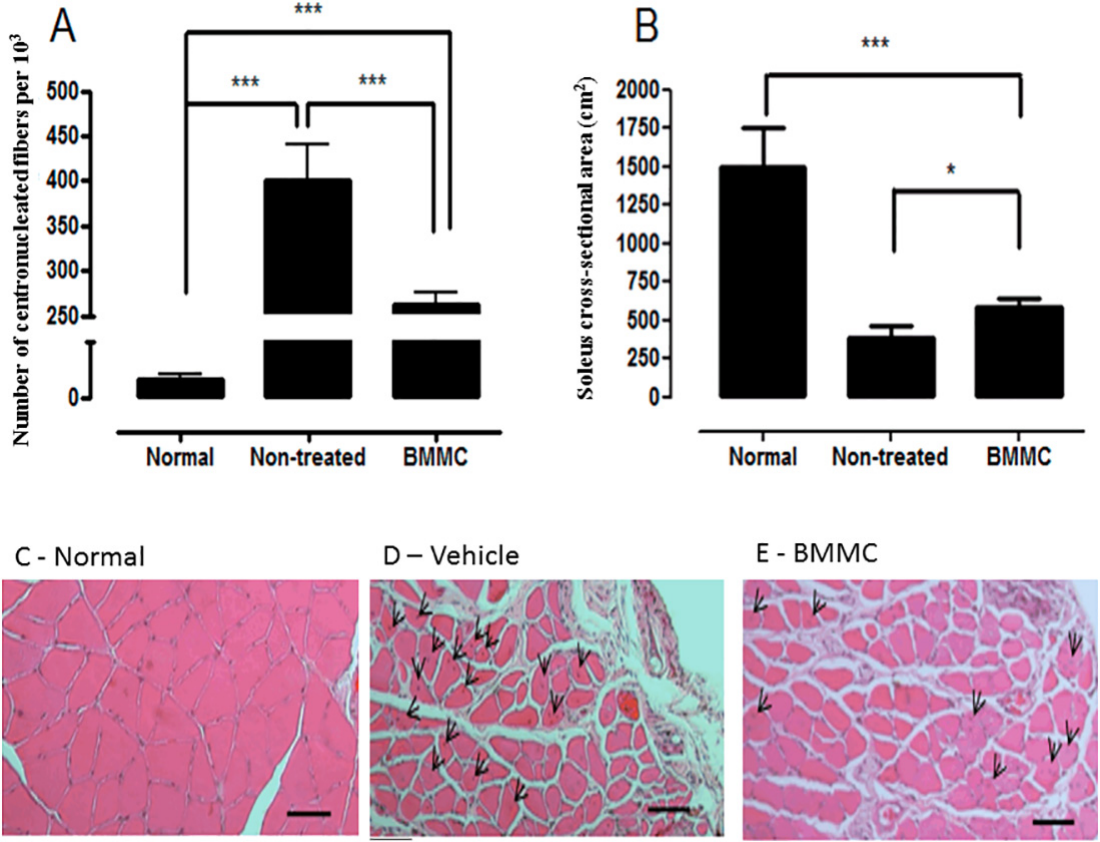
Centronucleated muscle fiber and cross-sectional area determination 28 days after muscle injury. (A) Number of centronucleated muscle fibers per 10^3^ fibers in normal (non-injured), vehicle- and BMMC-treated groups; (B) muscle fiber cross-sectional area in same groups as A; (C) Cross-sectional image a normal soleus muscle stained with H&E; (D) Same as in C but vehicle muscle is shown and (E) same as in C but BMMC soleus muscle is presented. Normal group values are from contralateral non-injured muscle. Values are means ± SD. *** p<0.001 * p<0.05. BMMC (n = 4) and vehicle-treated rats (n = 3). Centronucleated muscle fibers are shown by arrow reads.

At 28 days after injury, the BMMC-treated had fewer fibers with centrally located nuclei compared to non-treated rats (Fig 3A) suggesting a late stage of regeneration with more mature muscle fibers. Furthermore, BMMC increased muscle fiber cross-sectional area compared to non-treated muscles (580.1 ± 55.1 μm^2^ vs 390.0 ± 68.3 μm^2^, respectively; Fig 3B).

### Muscle and vessel markers in transplanted BMMC

**Fig 4:**
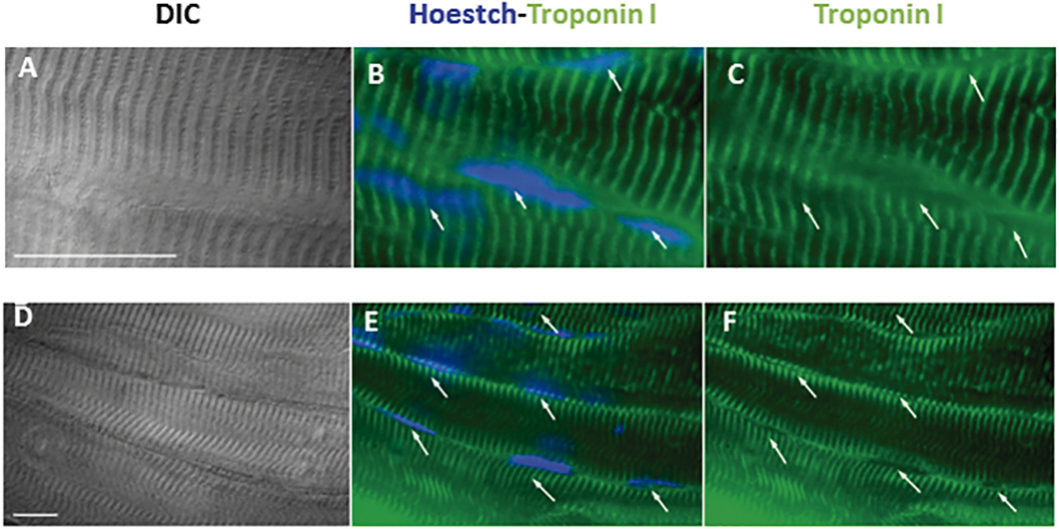
Skeletal muscle troponin immunolocalization in soleus muscle treated with BMMC 28 days after repeated injuries. (A) and (D) Differential interference contrast images; (B) and (E) overlay images of Hoestch (in blue) and troponin I (green); (C) and (F) troponin I staining alone. Green fluorescence indicates typical striated skeletal muscle labelling pattern while nuclei are shown by blue Hoescht staining. Arrows denote nuclei position. Bars correspond to 20μm.

**Fig 5:**
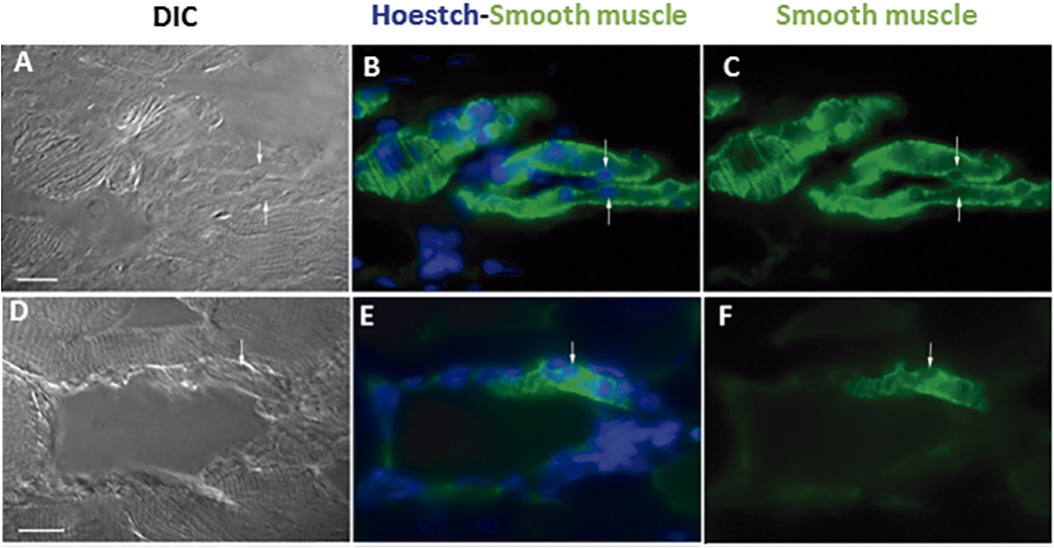
Immunolocalization of smooth muscle myosin as vessel marker in soleus muscle treated with BMMC 28 days after repeated injuries. A) and (D) Differential interference contrast images; (B) and (E) overlay images of Hoestch (in blue) and smooth muscle myosin (green) staining; (C) and (F) smooth muscle myosin alone. Green fluorescence indicates typical vessel shape labelling pattern while nuclei are shown by blue Hoescht staining. Arrows denote nuclei position. Bars correspond to 20μm.

One of the proposed mechanisms for cell therapy benefit is the differentiation of transplanted cells into host tissue cells, e.g. muscle fiber and/or vessel cells in skeletal muscle tissue. Thus, we stained skeletal muscle tissue with troponin I (Fig 4) or smooth muscle myosin (Fig 5) to determine the fate of the transplanted cells previously stained with Hoestch 33342 and found labeled cells in all treated skeletal muscle although in few muscle fibers and vessels. Merged images of Hoestch and troponin I staining indicate that donor cells engrafted skeletal muscle (Fig 4). Nuclei of transplanted cells were located either in the peripheral (Fig 4A–4C) or central position (Fig 4D–4F) in skeletal muscle fibers. Regarding the distribution of Hoescht-labeled nuclei in blood vessel structures, Fig 5 shows sectioned vessels harboring typical heavy myosin smooth muscle labelling pattern of the tunica media with concomitant nuclei Hoescht staining. A discontinuous labelling of the vessel smooth muscle marker can be readily noticed in the space occupied by the nuclear compartment indicating that the labelled nuclei could belong to the smooth muscle cells (Fig 5).

Altogether, immunostaining data suggest that transplanted BMMC could engraft into host muscle and express both myofiber and vessel markers. However, not all Hoestch-stained nuclei were present in skeletal muscle or vessel cells. We carefully counted 267 labeled nuclei of three BMMC-treated soleus and determine their position in the host tissue. Among the 267 stained nuclei, the majority (73%) was found in myofibers or smooth muscle cells (195 out of 267). On the other hand, 27% of the labeled nuclei were found to be in the interstitial space.

## Discussion

Our model of repeated injuries in rat soleus muscle markedly impaired muscle function and caused wide spread scar tissue formation. Maximal developed tension was reduced to about 66 and 30% of the normal value on day 14 and 28 after muscle injuries, respectively. In the cell treated group muscle tension development was significantly higher than in the vehicle group at both time points and recovery to control muscle tension values was observed 28 days after cell injection (Fig 1). This demonstrates that local injection of bone marrow mesenchymal cells (BMMC) significantly improves functional capacity of soleus muscle 14 and 28 days after re-injuries in comparison to vehicle-treated group. It is possible that longer observation times would result in complete recovery of tension development even in the vehicle group; this remains to be investigated and will allow distinction between an accelerated repair mechanism after cell therapy and a gain of function improvement induced by the cells.

Muscle injury and, mostly, re-injuries are a challenge for sports medicine and orthopedics [1,2]. Despite the great regenerative capacity of skeletal muscle, completely lacerated muscle recovers only approximately 50% of their strength and 80% of their ability to shorten [27]. Anti-inflammatory drugs, ultrasound therapy, ice and superficial heat do not target the main problem of muscle damage: cell loss. Cell transplantation suits this requirement and has been useful in muscle damage treatment. In the current work we injected the adherent fraction of bone marrow mononuclear cells, a population of CD34^-^/CD45^-^ cells [10,11], the BMMC, directly into damaged soleus muscles. Our group has shown that these adherent bone marrow cells are capable of differentiating in mesenchymal lineage such as adipogenic, osteogenic and chrondogenic [28]. To optimize retention, BMMC were injected in matrigel, which is a reconstituted basement membrane, containing predominantly laminin-1, collagen IV, and perlecan, a heparin sulfate proteoglican (Matrigel) that supports cells adhesion and can prevent cell loss due to diffusion [29].

BMMC have been reported to contribute to muscle regeneration [20] and improve functional capacity [12,19,30] after muscle lesion. More important, Palermo et al (2005) demonstrated that bone marrow cells migrate to skeletal muscle in response to physiologic stress [21]. The differentiation and fate of transplanted donor-derived cells in skeletal muscle repair is still a controversial issue. In contrast to Natsu et al (2004), we did find transplanted cells in host muscle tissue, as described by others [17,20,21,23,31–35]. Blue nuclei indicate that BMMC, previously stained with the Hoestch dye, were present in the damage tissue and some of these cells showed immunoreactivity for smooth muscle myosin and skeletal muscle troponin (Figs 4 and 5). Matziolis et al (2006) also performed a crunch model, but lesion was induced by only seven manual clamping one week before cell transplant. Unfortunately, they did not show any imuno and/or histological analisys [12]. However, we cannot exclude a paracrine effect of the transplanted cells inducing functional improvement. Given that the frequency of donor cells in host tissue 28 days after muscle cell injections is low, we cannot rule out other possible beneficial effects of BMMC treatment such as a paracrine mechanism. Indeed, we recently demonstrate that soluble factors secreted by BMMC improve cardiac function in infarcted rat hearts [25]. Also, we reported that mesenchymal cells from adipose tissue improve muscle function even with no cell found in the host tissue 4 weeks after injetion [13].

The functional improvement did not correlate with scar tissue formation. We found that injection of BMMC in crunched muscles did not decrease the amount of fibrosis and scar formation at the re-injured site (Table 1 and Fig 2). Despite its high regeneration capacity, muscle lesion always leads to extracellular matrix expansion, collagen deposition and formation of a fibrotic scar. This process is strictly related to the level of muscle contusion. Moreover, successive injuries would exacerbate scar formation. In this context, both mononuclear and mesenchymal cells form bone marrow did not reverse liver fibrosis [28,36] and cells from adipose tissue were not capable of decreasing fibrosis in SKM laceration model [13]. Accordingly, antifibrotic therapy has been tested after muscle injury [35,37–44] and was able to improve cell-based therapies [45–47].

During muscle regeneration, new skeletal muscle fiber exhibit their nuclei centrally located and then finally matures to periphery nucleated fibers [3]. Thus the number of fiber with centrally located nuclei imputes an immature muscle and/or in regeneration phase. The maturation constitutes the third and last phase of the degeneration/regeneration cycle and is characterized by fiber hypertrophy and gradual recovery functional properties of the muscle [3,48]. Treatment with BMMC increased soleus muscle cross-sectional area and decreased the number of fiber with centrally located nuclei suggesting a more mature phenotype of treated muscles. Therefore, increasing the pool of cells capable of differentiating into skeletal muscle cells is a promising tool to help in the treatment of serious and recidivous/recurrent injuries as in sports, aging and car accidents. Taken together, our results suggest that bone marrow injected cells might contribute to myotube formation process (repairing damaged muscle fibers) and/or form new muscle fibers regardless of fibrosis. Alternatively, as mentioned above, paracrine secretion of growth and angiogenic factors might result in improved function through hypertrophy and angiogenesis [49].

In conclusion, bone marrow stromal cells accelerate muscle regeneration and restore soleous muscle function despite scar tissue development in a severe re-injury protocol which is commonly found in recreational and elite sports due to insufficient regeneration.
